# Associations between Tissue Visfatin/Nicotinamide, Phosphoribosyltransferase (Nampt), Retinol Binding Protein-4, and Vaspin Concentrations and Insulin Resistance in Morbidly Obese Subjects

**DOI:** 10.1155/2013/861496

**Published:** 2013-12-03

**Authors:** Zeynep Goktas, Shannon Owens, Mallory Boylan, David Syn, Chwan-Li Shen, Debra B. Reed, Susan San Francisco, Shu Wang

**Affiliations:** ^1^Nutritional Sciences Program, College of Human Science, Texas Tech University, P.O. Box 41240, Lubbock, TX 79409-1240, USA; ^2^Texas Tech University Health Sciences Center, Lubbock, TX 79430, USA; ^3^Department of Pathology, Texas Tech University Health Sciences Center, Lubbock, TX 79430, USA; ^4^Center for Biotechnology and Genomics, Texas Tech University, Lubbock, TX 79409, USA

## Abstract

Visfatin/Nampt, vaspin, and retinol binding protein-4 (RBP-4) play an important role in insulin resistance. The objectives of this study were to measure visfatin/Nampt, vaspin, and RBP-4 concentrations in blood, liver, muscle, subcutaneous, omental, and mesenteric adipose tissues in morbidly obese subjects and investigate their relationship to insulin resistance. Blood and tissue samples were collected from 38 morbidly obese subjects during Roux-en-Y surgery. Insulin resistance biomarkers were measured using standard kits. Visfatin/Nampt, vaspin, and RBP-4 gene expression levels in tissues were measured using real-time PCR. Their protein concentrations in blood and tissues were measured using ELISA kits. Diabetic subjects had significantly higher homeostasis model of assessment-insulin resistance and age and lower blood HDL-cholesterol concentrations than nondiabetic and prediabetic subjects. Diabetic and prediabetic subjects had significantly higher blood concentrations of visfatin/Nampt and vaspin than nondiabetic subjects. Liver RBP-4 concentrations were positively associated with blood glucose concentrations. Blood insulin resistance biomarker levels were positively associated with visfatin/Nampt concentrations in omental adipose tissue and liver, and vaspin concentrations in mesenteric adipose tissue. In conclusion, the correlations of visfatin/Nampt, vaspin, and RBP-4 with insulin resistance are tissue dependent.

## 1. Introduction

Obesity is a multifactorial disease, which is linked to many chronic diseases including cardiovascular disease and type 2 diabetes [1, 2]. Many research studies indicate that obesity is a low-grade inflammatory condition [3, 4]. Expanded adipose tissue functions as an endocrine organ, which produces and secretes a number of hormones and cytokines known as adipokines [5, 6]. Visfatin/Nampt, vaspin, and retinol binding protein-4 (RBP-4) are novel adipokines and may play important roles in insulin resistance development. Increased visceral fat mass was positively associated with insulin resistance [7, 8].

Visfatin was identified as an adipokine in 2005 by Fukuhara and colleagues [9]. Early research suggested that this peptide adipokine produces insulin-mimetic effects by binding to the insulin receptors and activating downstream insulin signaling pathways [9–11]. However, due to failure to reproduce these data, Fukuhara and colleagues retracted their findings in 2007 [12]. The relationship between insulin resistance and blood or tissue visfatin/Nampt concentrations is not clear. Some studies indicate that blood visfatin/Nampt concentrations significantly correlate with insulin resistance or type 2 diabetes but not with body fat percentage or body mass index (BMI) [13–15]. Other studies demonstrate that the association between diabetes and blood visfatin/Nampt concentrations was not significant after adjusting for body mass index (BMI) and waist circumference [14, 16].

Vaspin is known as visceral adipose-tissue derived serpin [17]. Vaspin has some effects on increasing glucose uptake by adipocytes. After administrating vaspin to obese mice, glucose tolerance and insulin sensitivity were significantly improved [18]. Some studies demonstrated that blood vaspin concentrations were higher in diabetic subjects compared to nondiabetic subjects [19, 20]. However, the relationship between blood vaspin concentrations and insulin resistance is inconclusive [21].

Adipocytes and hepatocytes are major cells to produce retinol binding protein-4 (RBP-4) in humans. RBP-4 functions as retinol carrier protein in plasma. It also regulates glucose metabolism and insulin sensitivity [22]. Increased blood RBP-4 concentrations can reduce glucose uptake by muscle cells through inhibiting the expression and serine phosphorylation of insulin receptor substrate-1 (IRS-1) and phosphatidylinositol 3-kinase (PI3K) signaling pathway resulting in decreased insulin sensitivity [23]. Some studies demonstrated that glucose transporter-4 (GLUT4) protein expression in muscle cells was inversely correlated with plasma RBP-4 concentrations in obese subjects with impaired glucose tolerance or type 2 diabetes [22, 24]. In addition, increased blood RBP-4 concentrations can decrease GLUT4 expression in adipose tissue in obese subjects [25].

The relationship between insulin resistance and tissue adipokines, especially visfatin/Nampt, vaspin, and RBP-4, is not completely understood. The purpose of this study was to measure gene expression levels and protein concentrations of visfatin/Nampt, vaspin, and RBP-4 in liver, muscle, subcutaneous, omental, and mesenteric adipose tissues and their concentrations in blood collected from severely obese patients who underwent bariatric surgery. Furthermore, we investigated the relationship between insulin resistance and their gene expression and protein concentrations in the above five tissues.

## 2. Materials and Methods

### 2.1. Study Design and Subjects

This was a cross-sectional study of measuring visfatin/Nampt, vaspin, and RBP-4 gene expression and protein levels in liver, muscle, subcutaneous, omental, and mesenteric adipose tissue and their plasma concentrations in severely obese patients. Subjects were recruited from the Advanced Bariatric Surgery Center in Lubbock, TX, by a bariatric surgeon and a clinical dietitian. Thirty-eight subjects who were planned for Roux-en-Y surgery agreed to participate in the study. All subjects signed a consent form. Subjects included 12 males and 26 females with mean age 46 y (range 18–60 y), mean weight 142 kg (range 96–243 kg), and mean BMI 49 kg/m^2^ (range 37–81 kg/m^2^). The exclusion criteria were as follows: previous bariatric surgery, any autoimmune or inflammatory disease, medical treatment for type 2 diabetes, or insulin resistance. Fasting blood was collected in EDTA-coated collection tubes in the operation room before starting the surgery. The blood analyses were completed on every patient before surgery started. Excess blood samples were centrifuged immediately for 25 minutes at 11,000 ×g at 4°C. Plasma was aliquoted and collected in new tubes and stored at −80°C. The surgeon collected liver (a wedge biopsy, 0.5–1 g), rectus abdominus muscle (1 g), subcutaneous, omental, and mesenteric adipose tissue (1–5 g) during the surgery. Tissues were washed three times in Krebs Ringer solution (124 mM sodium chloride, 2.5 mM potassium chloride, 1.25 mM sodium phosphate monobasic, 26 mM sodium bicarbonate, 2 mM calcium chloride, 1 mM magnesium sulfate, and 10 mM glucose), immersed into liquid nitrogen immediately, and stored at −80°C until subsequent analyses. All study procedures were approved by the institutional review board at Texas Tech University Health Sciences Center.

### 2.2. Blood Lipid and Insulin Resistance Biomarkers

Subjects' age, weight, BMI, gender, fasting blood concentrations of glucose, triglyceride, total cholesterol and HDL-cholesterol, and blood hemoglobin A1c (HbA1c) levels were measured using standard methods at Texas Tech University Medical Center. Plasma insulin was analyzed using commercially available insulin human direct enzyme-linked immunosorbent assay (ELISA) kit (Invitrogen, Grand Island, New York). Homeostasis model of assessment-insulin resistance (HOMA-IR) was calculated as fasting blood glucose (mmol/L) × fasting plasma insulin (mU/L)/22.5.

### 2.3. RNA Extraction and Reverse Transcription

Adipose tissues were homogenized using QIAzol Lysis Reagent (Qiagen, San Jose, California) with a PowerGen Model 500 Homogenizer (Fisher Scientific, Pittsburgh, Pennsylvania). Adipose tissue RNA was extracted using RNeasy Lipid Mini Kit (Qiagen, San Jose, California). Liver and muscle tissues were homogenized in TRIzol Reagent (Life Technologies, Grand Island, New York) uisng a PowerGen Model 500 Homogenizer (Fisher Scientific, Pittsburgh, Pennsylvania). After homogenization, liver and muscle RNA was extracted following a standard Trizol RNA extraction protocol (Life Technologies, Grand Island, New York). The concentration of total RNA was measured using Thermo Scientific NanoDrop 2000 spectrophotometer (Thermo Scientific, Marietta, Ohio).

Single strand cDNA was synthesized from RNA using SuperScript III reverse-transcriptase (Invitrogen, Grand Island, New York). RNA (1 *μ*g) was reverse transcribed in a 20 *μ*L final volume consisting of UltraPure DNase/RNase-Free Water (Invitrogen, Grand Island, New York), random primers (Invitrogen, Grand Island, New York), PCR nucleotide mix (Roche, Indianapolis, Indiana), SuperScript III reverse transcriptase (Invitrogen, Grand Island, New York), and RNaseOUT recombinant RNase inhibitor (Invitrogen, Grand Island, New York) following SuperScript III reverse transcriptase manufacturer's instruction.

### 2.4. Real-Time PCR

cDNA levels of the genes of interest were determined using power SYBR green master mix (Applied Biosystems, Grand Island, New York) on an Eppendorf Mastercycler Ep Realplex (Fisher Scientific, Pittsburgh, Pennsylvania). Primers were designed at the exon-exon junctions to eliminate genomic DNA amplification, using Primer Express 1.0 (Applied Biosystems, Grand Island, New York) and purchased from Integrated DNA Technologies (IDT, San Diego, California). Primers used to amplify visfatin/Nampt were 5′-GGTTCTGGTGGAGGTTTGCTAC-3′ (forward) and 5′-GAAGACGTTAATCCCAAGGCC-3′ (reverse), and vaspin primers were 5′-AATAAGTGTTGCTGCCCTGGTG-3′ (forward) and 5′-TGCCGCCAGAACTTTCCAT-3′ (reverse), and RBP-4 primers were 5′-TACTCCTTCGTGTTTTCCCGG-3′ (forward) and 5′-TAACCGTTGTGGACGATCAGC-3′ (reverse), and beta (*β*)-actin primers were 5′-CTTTTCCAGCCTTCCTTCTTGG-3′ (forward) and 5′-CAGCACTGTGTTGGCATAGAGG-3′ (reverse). *β*-Actin was used as an endogenous control. Primer amplification efficiency and specificity were verified for each set of primers. The real-time PCR reaction condition was 95°C for 10 minutes, 40 cycles of 95°C for 15 seconds and 60°C for 1 minute, and 1 cycle of dissociation stage (95°C for 15 seconds, 60°C for 30 seconds at and 95°C for 15 seconds). All samples were run in duplicate and the average values were used. Relative quantification was calculated using the ΔΔCt method.

### 2.5. Protein Extractions

About 80–100 mg of adipose tissues was homogenized using a PowerGen Model 500 homogenizer (Fisher Scientific, Pittsburgh, Pennsylvania) in radioimmunoprecipitation assay (RIPA) buffer (25 mmol/L Tris HCl, 10 mmol/L sodium orthovanadate, 100 mmol/L sodium fluoride, 50 mmol/L sodium pyrophosphate tetrabasic, 10 mmol/L ethylene glycol tetraacetic acid (EGTA), 10 mmol/L ethylenediaminetetraacetic acid (EDTA), and 1% IGEPAL) with protease inhibitors. All the above components were purchased from Sigma-Aldrich Chemical Co. (St. Louis, Missouri). About 50 mg of liver or muscle tissues was homogenized uisng a PowerGen Model 500 homogenizer (Fisher Scientific, Pittsburgh, Pennsylvania) in CelLytic reagent (Sigma-Aldrich Co., St. Louis, Missouri) containing protease inhibitors (Sigma-Aldrich Co., St. Louis, Missouri). After homogenization, the tissue lysates were centrifuged at 15,000 rpm for 30 minutes at 4°C. Upper aqueous phases of the lysates were collected and aliquoted into several new tubes as total protein extracts.

### 2.6. ELISA

Visfatin/Nampt, vaspin, and RBP-4 concentrations in plasma and tissue extracts were analyzed using commercially available ELISA kits (Adipogen, Seoul, South Korea) according to the manufacturer's instructions. After protein extraction, the tissues were dried and digested using 1 N NaOH. Cellular protein concentrations were measured using a bicinchoninic acid (BCA) assay kit (Pierce, Rockford, lllinois). Plasma and tissue adipokine concentrations were expressed as ng/mL and ng/mg of protein, respectively.

### 2.7. Statistical Analyses

Statistical analyses were performed using the computer program PSAW 18.0 (IBM, USA). Levene's test of normality was used to test the distribution of variables. One-way ANOVA was used for comparison of more than two group means. For post hoc analysis, least significant difference (LSD) was used with equal variances and Games-Howell test was used with unequal variances. Analysis of covariance was used to compare more than two group means while controlling the covariates such as diabetic classification. Correlations among variables were tested with Pearsons correlation coefficient after adjusting the data for BMI. Data were presented as means ± standard deviation. Differences were considered significant at *P* < 0.05.

## 3. Results

### 3.1. Subject Characteristics, Blood Lipid, and Insulin Resistance Biomarkers

All subjects were morbidly obese with an average BMI of 49.3 ± 8.76 kg/m². Based on blood HbA1c levels following the NIH diabetic classification guidelines, subjects were divided into nondiabetic (*n* = 13), prediabetic (*n* = 12), and diabetic (*n* = 13) groups (Table 1). Diabetic subjects had significantly higher blood glucose concentrations, HbA1c levels, homeostasis model of assessment-insulin resistance (HOMA-IR) and age, and lower blood HDL-cholesterol concentrations than nondiabetic and prediabetic subjects (Table 1). Diabetic subjects had lower BMI and body weight than nondiabetic and prediabetic subjects, but they did not reach statistical significance (Table 1).

### 3.2. Blood Concentrations of Visfatin/Nampt, Vaspin, and RBP-4

Prediabetic and diabetic subjects had significantly higher blood visfatin/Nampt and vaspin concentrations than nondiabetic subjects (Table 2). Blood RBP-4 concentrations were similar among three groups (Table 2). Neither blood visfatin/Nampt, RBP-4, or vaspin correlated with HOMA-IR, blood glucose, or HbA1c (%) levels.

### 3.3. Visfatin/Nampt Tissue Expression and Its Association with Insulin Resistance

**Figure 1:**
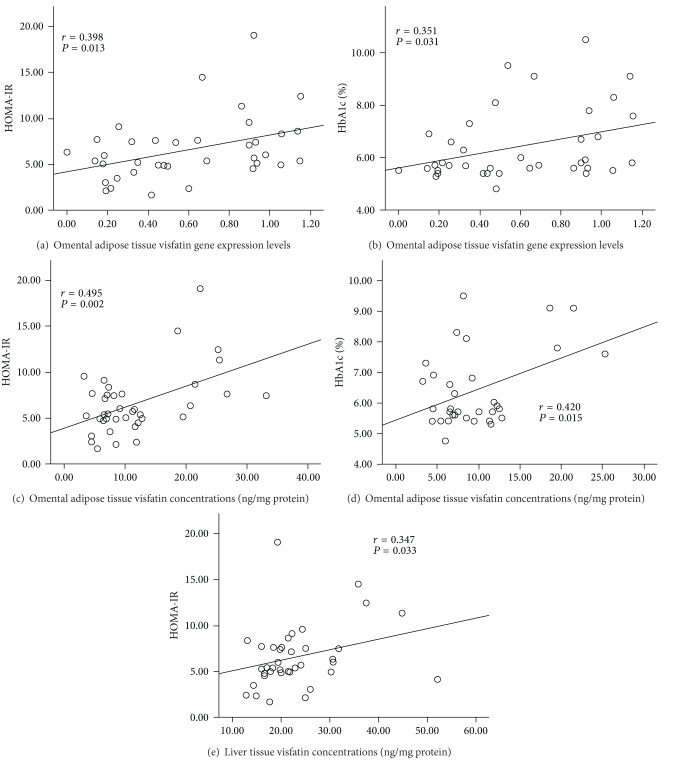
Correlation between tissue visfatin/Nampt gene or protein expression and blood insulin resistance biomarkers.

Omental visfatin/Nampt gene expression levels were positively correlated with HOMA-IR (Figure 1(a)) and blood HbA1c levels (Figure 1(b)). Omental visfatin/Nampt protein concentrations were positively correlated with HOMA-IR (Figure 1(c)) and blood HbA1c levels (Figure 1(d)), which was accompanied with the significantly higher omental visfatin/Nampt protein concentrations in diabetic compared to nondiabetic and prediabetic subjects (Table 3). Liver had the highest visfatin/Nampt concentrations among five tissues. Omental visfatin/Nampt protein concentrations were significantly correlated with its mRNA levels (see Supplementary table in Supplementary Material available online at http://dx.doi.org/10.1155/2013/861496). Liver visfatin/Nampt protein concentrations were positively correlated with HOMA-IR (Figure 1(e)). Visfatin/Nampt in other tissues did not correlate with insulin resistance.

### 3.4. RBP-4 Tissue Expression and Its Association with Insulin Resistance

**Figure 2:**
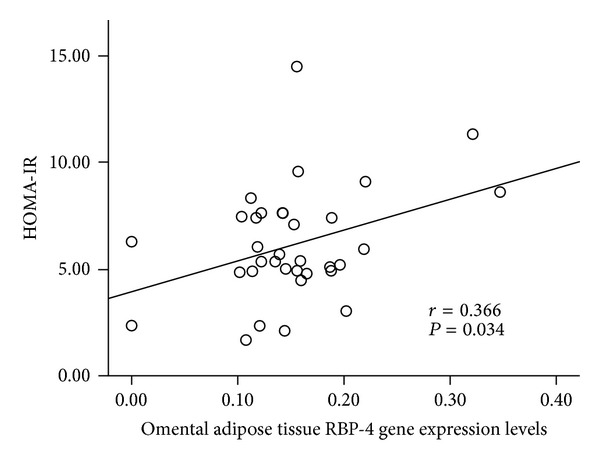
Correlation between tissue RBP-4 gene or protein expression and blood insulin resistance biomarkers.

Omental adipose tissue RBP-4 gene expression levels were positively correlated with HOMA-IR (Figure 2). Liver had the highest RBP-4 concentrations, followed by muscle, subcutaneous, omental, and mesenteric adipose tissues in a decreased order (Table 3). RBP-4 concentrations in other tissues did not correlate with insulin resistance.

### 3.5. Vaspin Tissue Expression and Its Association with Insulin Resistance

**Figure 3:**
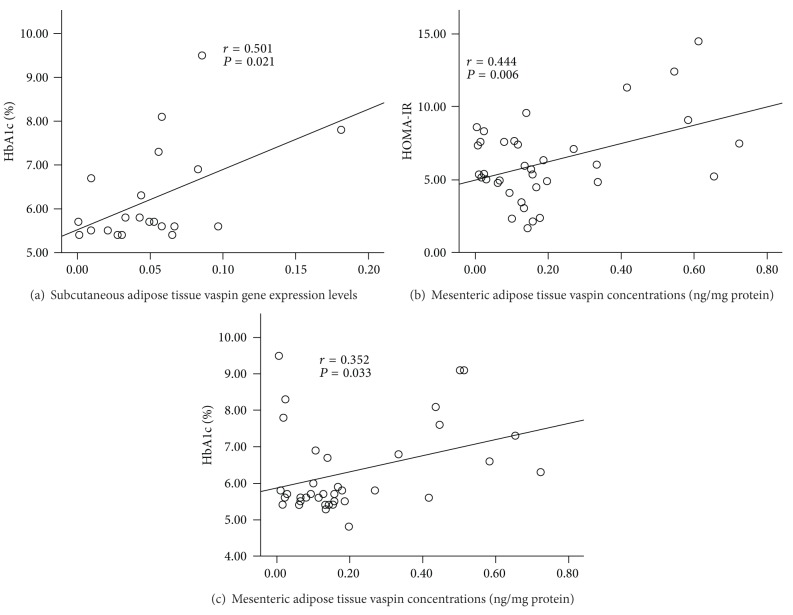
Correlation between tissue vaspin gene or protein expression and blood insulin resistance biomarkers.

Subcutaneous adipose tissue vaspin gene expression levels were positively correlated with blood HbA1c levels (Figure 3(a)). Mesenteric adipose tissue vaspin protein concentrations were positively correlated with HOMA-IR (Figure 3(b)) and blood HbA1c levels (Figure 3(c)). Diabetic subjects had about 3-fold higher mesenteric vaspin concentrations than nondiabetic and prediabetic subjects (Table 3). But the difference was not significant, due to higher standard deviations. Liver had the highest vaspin concentrations among 5 tissues, and prediabetic and diabetic subjects had significantly higher liver vaspin concentrations than nondiabetic subjects (Table 3). But liver vaspin did not correlate with any insulin resistance biomarkers.

## 4. Discussion

The current study was the first study to measure gene expression levels and protein concentrations of visfatin/Nampt, vaspin, and RBP-4 in liver, muscle, subcutaneous, omental, and mesenteric adipose tissues and their protein concentrations in plasma and to investigate the relationship between insulin resistance and their expression in blood and tissues in morbidly obese subjects.

Visfatin/Nampt was originally discovered by Samal and colleagues in 1994 as Pre-B-cell colony-enhancing factor (PBEF) in liver, muscle, and bone marrow [26]. Later studies showed that visfatin/Nampt was also produced by a variety of cells including lymphocytes, monocytes, neutrophils, hepatocytes, and adipocytes [27]. Increased visfatin/Nampt concentrations in plasma have been reported in obese subjects [14]. However, it is unknown whether the increased visfatin/Nampt concentrations in blood or tissues are related to insulin resistance. In the current study, diabetic and prediabetic subjects had higher blood visfatin/Nampt concentrations than nondiabetic subjects, which is consistent with other studies [13–16, 28, 29]. This is the first study to measure visfatin/Nampt protein concentrations in liver, muscle, and three adipose depots and correlate their concentrations with insulin resistance. Liver had the highest visfatin/Nampt protein concentrations among five collected tissues. Liver visfatin/Nampt protein concentrations had a positive association with HOMA-IR, but liver visfatin/Nampt concentrations were similar among three groups. Omental adipose tissue had higher visfatin/Nampt protein concentrations than subcutaneous and mesenteric adipose tissues. Furthermore, visfatin/Nampt protein concentrations and gene expression levels in omental adipose tissue were positively correlated with HOMA-IR and blood HbA1c levels. Diabetic subjects had significantly higher visfatin/Nampt protein concentrations in omental adipose tissue than nondiabetic and prediabetic subjects. Diabetic subjects had impaired glucose tolerance, which may induce visfatin/Nampt production from omental adipose tissue via a compensatory mechanism. Impaired glucose tolerance in diabetic subjects might stimulate visfatin/Nampt production from omental adipose tissue to compensate for the insulin resistance [30]. We could not find any correlation between BMI and visfatin/Nampt expression levels in all five tissues. These data suggest that BMI may not be effective in inducing visfatin/Nampt production in omental adipose tissue. Overall, these findings suggest that visfatin/Nampt in omental adipose tissue may play an important role in insulin resistance.

Many studies demonstrate that RBP-4 is a pro-inflammatory adipocytokine [22, 25]. It is secreted predominantly by adipocytes and hepatocytes in humans [22]. Since increased RBP-4 plasma levels were shown in GLUT4 knockout mice, many research studies have investigated the relationship between RBP-4 and insulin resistance [24, 25]. Even though some studies demonstrated that plasma RBP-4 levels positively correlated with insulin resistance in obese subjects with impaired glucose tolerance or type 2 diabetes [24], most of studies suggested that blood RBP-4 concentrations were not associated with insulin resistance [31]. We found that nondiabetic, prediabetic, and diabetic morbidly obese subjects had similar blood RBP-4 concentration in this study. The association between blood RBP-4 concentrations and insulin resistance is influenced by many confounders including age, BMI, disease status, nutrition status, genetic factors, and others. More studies with large sample size are required to adjust these confounders. Numerous research studies have demonstrated that RBP-4 gene expression levels in visceral adipose tissue are positively correlated with insulin resistance [24, 25, 32]. In our current study, we found that omental, not mesenteric, adipose tissue RBP-4 gene expression levels were positively correlated with HOMA-IR and fasting blood glucose concentrations. Furthermore, liver had the highest RBP-4 concentrations among all five tested tissues; however, liver RBP-4 protein concentrations did not correlate with fasting blood glucose concentrations, HOMA-IR, and blood HbA1c levels. The data suggest that liver RBP-4 might not correlate with insulin resistance, but more studies are required to generate a solid conclusion.

Vaspin is mainly expressed in human adipose tissues [33]. It has been considered as a visceral adipokine [34]. We also demonstrated that liver had the highest vaspin concentrations among 5 tested tissue. But liver vaspin did not correlate with insulin resistance. Even though mesenteric adipose tissue had the lowest vaspin concentrations among the three collected adipose tissues, mesenteric adipose tissue vaspin protein concentrations were positively correlated with HOMA-IR and blood HbA1c levels. Additionally, diabetic subjects had about 3-fold higher mesenteric adipose tissue vaspin concentrations than nondiabetic and prediabetic subjects. Insulin resistance may stimulate vaspin production through a compensatory mechanism in diabetic subjects. Vaspin can increase glucose uptake by adipocytes [21]. In the case of insulin resistance or impaired glucose tolerance, vaspin production in mesenteric adipose tissues might be increased in order to enhance the glucose uptake as a counter action to insulin resistance [20, 35]. Some research studies suggest that plasma vaspin levels are positively correlated with insulin resistance in diabetic subjects [32, 34, 35]. In the current study, diabetic and prediabetic subjects had higher plasma vaspin concentrations than nondiabetic subjects, which is consistent with other published studies [19, 20]. The association between plasma vaspin levels and BMI in obese subjects is contradictory [35, 36]. In our study, vaspin protein concentrations or gene expression levels in five tissues or plasma did not correlate with BMI or body weight. Some studies demonstrated that the positive correlation between plasma vaspin concentrations and BMI is more dominant in type 2 diabetes or insulin resistant subjects [20, 35, 36]. Increased plasma vaspin concentrations might be caused by insulin resistance rather than the high BMI [20, 35, 36]. The data indicate that mesenteric vaspin had an association with insulin resistance, and vaspin expression and production are affected by insulin resistance rather than BMI or obesity.

## 5. Conclusions

In summary, even though visfatin/Nampt, vaspin, and RBP-4 are called adipokines, liver had higher concentrations than adipose tissues. The correlations of visfatin/Nampt, vaspin, and RBP-4 with insulin resistance are tissue dependent. Insulin resistance was significantly associated with omental adipose tissue visfatin/Nampt and mesenteric adipose tissue vaspin concentrations in morbidly obese subjects. Liver RBP-4 and visfatin/Nampt might also be associated with insulin resistance, but more studies are required to further investigate and confirm the association.

## Supplementary Material

Supplementary Table: We compared visfatin, vaspin and RBP-4 protein concentrations and gene expression levels among all the tissues and plasma to see the correlations. Plasma visfatin levels positively correlated with visfatin muscle gene expression levels (*r* = 0.394, *p* = 0.047). Plasma RBP-4 levels did not correlate with any tissue RBP-4 protein concentrations and gene expression levels. Plasma vaspin levels positively correlated with muscle tissue vaspin protein concentration (*r* = 0.538, *p* < 0.001). Although we found some correlations among different tissue protein and gene expression levels, there was no correlation between visfatin, vaspin and RBP-4 protein concentrations and gene expression levels within the same tissue.

## Figures and Tables

**Table 1:** Characteristics of nondiabetic, prediabetic, and diabetic subjects^¥^.

	Nondiabetic (*n* = 13)	Prediabetic (*n* = 12)	Diabetic (*n* = 13)
Age	39 ± 12^b^	46 ± 10^b^	54 ± 9^a^
Weight (kg)	144.8 ± 36.49	148.5 ± 27.26	133.9 ± 23.68
BMI (kg/m²)	49.6 ± 11.72	51.8 ± 6.89	46.9 ± 6.59
Blood glucose (mg/dL)	92.6 ± 7.95^b^	97.6 ± 15.34^b^	169.4 ± 64.28^a^
HbA1c (%)	5.4 ± 0.21^b^	5.8 ± 0.21^b^	8.0 ± 1.22^a^
Plasma insulin (*µ*IU/mL)	27.5 ± 11.70	23.6 ± 6.85	30.3 ± 15.31
HOMA-IR	5.5 ± 2.54^b^	4.9 ± 1.76^b^	11.4 ± 10.15^a^
Triglyceride (mg/dL)	136.0 ± 38.17	133.1 ± 55.25	200.2 ± 111.59
TC (mg/dL)	191.3 ± 32.28	185.8 ± 75.38	177.5 ± 46.10
HDL-C (mg/dL)	47.6 ± 10.95^b^	43.8 ± 11.42^b^	36.5 ± 6.39^a^

^*¥*^Data are presented as means ± SD.

Means within a row without a common letter differ, *P* < 0.05.

BMI: body mass index; HbA1c: hemoglobin A1c; HOMA-IR: homeostasis model of assessment-insulin resistance; HDL-C: high density lipoprotein-cholesterol; TC: total cholesterol.

**Table 2:** Visfatin/Nampt, vaspin, and RBP-4 protein concentrations in plasma^¥^.

	Nondiabetic (*n* = 13)	Prediabetic (*n* = 12)	Diabetic (*n* = 13)
Visfatin/Nampt (ng/mL)	17.4 ± 8.7^b^	20.6 ± 12.3^a^	20.6 ± 12.3^a^
RBP-4 (ng/mL)	38.5 ± 14.3	43.8 ± 10.0	36.9 ± 14.3
Vaspin (ng/mL)	0.91 ± 0.2^b^	2.59 ± 0.5^a^	2.44 ± 0.2^a^

^*¥*^Data are presented as means ± SD.

Means within a row without a common superscript differ, *P* < 0.05.

RBP-4: retinol binding protein-4.

**Table 3:** Visfatin/Nampt, vaspin, and RBP-4 protein concentrations in different tissues^ ¥^.

Tissues (ng/mg protein)	Normal (*n* = 13)	Pre-Diabetic (*n* = 12)	Diabetic (*n* = 13)
Visfatin/Nampt			
Subcutaneous adipose	11.3 ± 13.01^b^	10.5 ± 10.11^b^	14.8 ± 17.42^b^
Omental adipose	11.5 ± 7.71^b^	11.4 ± 7.42^b^	17.5 ± 23.64^c∗^
Mesenteric adipose	10.5 ± 9.44^b^	16.6 ± 17.23^b^	11.7 ± 9.36^b^
Liver	24.0 ± 7.85^a^	22.5 ± 10.71^a^	22.8 ± 7.50^a^
Muscle	7.5 ± 4.81^b^	12.4 ± 11.60^b^	9.9 ± 8.77^b^

RBP-4			
Subcutaneous adipose	441.2 ± 83.75^c^	408.5 ± 49.11^c^	437.1 ± 63.37^c^
Omental adipose	421.4 ± 67.46^c^	420.0 ± 62.44^c^	442.2 ± 65.96^c^
Mesenteric adipose	353.9 ± 45.92^d^	314.2 ± 49.40^d^	348.3 ± 41.75^d^
Liver	11880.2 ± 1008^a^	11744.9 ± 791^a^	11248.1 ± 1209^a^
Muscle	638.2 ± 436.20^b^	768.4 ± 443.50^b^	530.4 ± 238.4^b^

Vaspin			
Subcutaneous adipose	0.66 ± 0.45^b^	0.09 ± 0.01^b^	0.90 ± 0.46^b^
Omental adipose	0.57 ± 0.16^ab^	2.26 ± 2.07^ab^	1.07 ± 0.36^ab^
Mesenteric adipose	0.23 ± 0.09^b^	0.17 ± 0.01^b^	0.67 ± 0.46^b^
Liver	1.41 ± 0.18^a^	3.05 ± 0.91^a∗^	2.17 ± 0.45^a∗^
Muscle	0.35 ± 0.05^b^	1.38 ± 0.39^b^	0.47 ± 0.08^b^

^*¥*^Data are presented as means ± SD.

*Means are significantly high within a row, *P* < 0.05.

^abcd^Means within a column for an adipokine without a common superscript differ, *P* < 0.05.

RBP-4: retinol binding protein-4.
