# Supplementary material for: Associations between Tissue Visfatin/Nicotinamide, Phosphoribosyltransferase (Nampt), Retinol Binding Protein-4, and Vaspin Concentrations and Insulin Resistance in Morbidly Obese Subjects
Source: Mediators Inflamm. 2013 Dec 3;2013:861496. doi: 10.1155/2013/861496 (PMC3866788; doi:10.1155/2013/861496)
Supplement: Supplementary file 1 — Supplementary Table: We compared visfatin, vaspin and RBP-4 protein concentrations and gene expression levels among all the tissues and plasma to see the correlations. Plasma visfatin levels positively correlated with visfatin muscle gene expression levels (r = 0.394, p = 0.047). Plasma RBP-4 levels did not correlate with any tissue RBP-4 protein concentrations and gene expression levels. Plasma vaspin levels positively correlated with muscle tissue vaspin protein concentration (r = 0.538, p < 0.001). Although we found some correlations among different tissue protein and gene expression levels, there was no correlation between visfatin, vaspin and RBP-4 protein concentrations and gene expression levels within the same tissue. [file 861496.f1.doc]

**Supplementary table: Correlations of visfatin, RBP-4 and vaspin within tissues and blood. ^€^, ***

| **Visfatin** | | | r | P value | | |
| --- | --- | --- | --- | --- | --- | --- |
| Plasma protein vs Muscle mRNA | | | 0.394 | 0.047 | | |
| Mesenteric protein vs. Subcutaneous protein | | | 0.443 | 0.005 | | |
| Omental protein vs. Omental mRNA | | | 0.341 | 0.036 | | |
| Omental mRNA vs. Liver mRNA | | | -0.325 | 0.047 | | |
| Omental mRNA vs. Muscle mRNA | | | -0.400 | 0.043 | | |
| Mesenteric mRNA vs. Muscle mRNA | | | 0.492 | 0.011 | | |
| Mesenteric mRNA vs. Liver mRNA | | | 0.548 | <0.001 | | |
| **RBP-4** | | r | | | P value | |
| No plasma-tissue correlation | |  | | |  | |
| Subcutaneous protein vs. Omental protein | | 0.355 | | | 0.029 | |
| Omental protein vs. Mesenteric protein | | 0.432 | | | 0.007 | |
| Liver protein vs. Omental protein | | -0.358 | | | 0.027 | |
| **Vaspin** | | | r | P value | | |
| Plasma protein vs. Muscle protein | | | 0.538 | <0.001 | | |
| Subcutaneous mRNA vs. Omental mRNA | | | 0.580 | 0.006 | | |
| Omental mRNA vs. Mesenteric mRNA | | | 0.994 | <0.001 | | |
| Liver protein vs. Omental protein | | | 0.393 | 0.015 | | |
|  | ^€^ Data analyzed by Pearson Correlation. This table only lists statistically significant correlation data.  * Correlation is significant at p < 0.05. | | | | |  |
